# Screening and Initiating Supportive Care in Patients With Heart Failure

**DOI:** 10.3389/fcvm.2019.00151

**Published:** 2019-10-22

**Authors:** Quynh Nguyen, Kaiming Wang, Anish Nikhanj, Dale Chen-Song, Ingrid DeKock, Justin Ezekowitz, Mehrnoush Mirhosseini, Bibiana Cujec, Gavin Y. Oudit

**Affiliations:** ^1^Division of Cardiology, Department of Medicine, Faculty of Medicine and Dentistry, Mazankowski Alberta Heart Institute, University of Alberta, Edmonton, AB, Canada; ^2^Division of Palliative Care Medicine, Department of Oncology, University of Alberta, Edmonton, AB, Canada

**Keywords:** heart failure, supportive care, palliative care, KCCQ, quality of life

## Abstract

**Background:** Patients with heart failure (HF) experience a major symptom burden and an overall reduction of quality of life. However, supportive care (SC) remains an under-utilized resource for these patients. Among the many existing barriers to integrating SC into routine care, identifying patients with SC needs remains challenging. The Kansas City Cardiomyopathy Questionnaire (KCCQ) is an important predictor of SC needs in patients with HF.

**Methods and Results:** We used the shortened version KCCQ-12 as a screening tool for SC need in our ambulatory HF patient population using a KCCQ-12 summary score of <29 as the cut-off. Of the 456 patients who completed the KCCQ-12, 41 (9%) were predicted to have SC needs. Demographics, medical history, biochemical parameters, echocardiographic assessment and medical treatment were similar between the two groups of patients. However, patients with KCCQ-12 <29 were more symptomatic based on both New York Heart Association (NYHA) classification and American Heart Association (AHA) staging with a higher prevalence of depression. We established a multidisciplinary SC clinic and the profile and outcomes of patients with SC needs that were referred and followed at our SC clinic were also evaluated. Twenty-three patients were referred to our SC clinic: 2 died before being seen, 1 refused SC and 20 received SC. Of these 20 patients, 11 died and 9 are currently being followed. Median survival after starting the SC clinic is 3 months. In the original SC cohort of 23, 17 patients had available KCCQ-12 summary scores. However, only 6 out of 17 (35%) had KCCQ-12 scores <29, indicating the need for additional assessment tools in this patient population.

**Conclusions:** The magnitude of unmet supportive care needs in patients with HF is significant. While the KCCQ-12 questionnaire is a useful tool to identify patients with SC, serial clinical evaluation, establishment of a SC clinic and prompt referral are essential for patients needing supportive care.

## Introduction

Heart failure (HF) is a complex syndrome characterized by the structural and/or functional impairment of heart function in a setting of multiple co-morbidities. Patients with HF often have long, frequent hospitalizations and high rates of hospital readmission (1–4). HF remains a common condition in North America, with an estimated 50% of the population expected to interact with an HF patient (5, 6). Patients with HF experience a major symptom burden, including dyspnea, pain, fatigue, poor appetite, anxiety, depression, and an overall impairment of quality of life (7, 8). For caregivers, the stress of supporting a loved one with a complex condition such as HF can be overwhelming (9).

Emerging evidence suggests that patients with HF would benefit from supportive care (SC); however, the magnitude of unmet SC in patients with HF remains high (3, 8, 10–12). Indeed, while SC is well integrated into the routine care of patients with advanced cancer (13–15), SC is only utilized in a small fraction of patients with HF despite recommendations of guidelines (7, 16). Referrals are often made during the last few months or weeks of life when death is imminent, thereby reducing the benefit of SC for patients with HF and their families (3, 17, 18). Among the many barriers to the integration of SC in cardiology, identifying patients who should be referred to SC appears to be the most challenging (3, 17). When tested in randomized trials, strategies for integrated SC into HF care is efficacious across several domains (19).

Patient-reported outcome measures are important independent outcomes in HF care and are predictive of clinical outcomes (20–22). The Kansas City Cardiomyopathy Questionnaire (KCCQ) is a valid, sensitive, disease-specific health status measure for patients with HF (22, 23). Various patient-reported outcome measures were previously evaluated for their predictive power in identifying patients with HF who should receive SC, and the KCCQ was found to be particularly predictive (22–25). A KCCQ summary score of <29 was found to be the strongest predictor of SC needs (26). Here, we investigated the utility of the KCCQ-12 to predict SC needs in an ambulatory HF patient population and secondly, described the impact of a multidisciplinary model of SC integrated into a contemporary HF clinic.

## Methods

### Study Patients and Protocol

The study protocol was reviewed and approved by the Health Research Ethics Board at the University of Alberta. All patients who participated in the study provided written informed consent. In the Heart Function Clinic (HFC) at the Mazankowski Alberta Heart Institute, patients were screened for study inclusion from February 6, 2018 to October 2, 2018. Details on the HFC have been previously described (27, 28). The multidisciplinary SC program is a novel initiative at the HFC. Patients were referred based on a combination of clinical judgment from physicians and patient clinical parameters. Eligible patients are referred to a community palliative care physician who performs a formal assessment and connects patients to available resources. The HFC coordinates the delivery of individualized patient care that aligns with the patient's needs. Patients were considered eligible for inclusion if they were above 18 years of age, have been previously diagnosed with HF by a cardiologist, and have provided written informed consent in accordance with the Declaration of Helsinki. Patients were excluded if they were unable or unwilling to provide written informed consent.

Patients with HF filled out the KCCQ-12 during study enrolment visit. Patient information and medical history were collected through interview with a study team member. Other relevant information, including medications, hospitalizations for HF, and any related outcome events (stroke, myocardial infarction, kidney, or liver failure) were also recorded. Information that could not be obtained directly from patients were gathered via review of medical records from electronic databases maintained by Alberta Health Services. American Heart Association (AHA) staging of HF was assessed according to the current guideline (29). Information on medications and co-morbidities were crosschecked with physical encounter letters from patient clinic visits to ensure data entry accuracy.

### Patient-Reported Outcome Measure

Study patients completed the KCCQ-12 with questions targeting four domains: physical limitation, symptom frequency, social limitation, and quality of life (23). KCCQ-12 summary scores were derived in the same manner as is done for the full KCCQ. Scores for each of the four domains were transformed to a 0 to 100 range by subtracting the lowest possible scale score, dividing by the range of the scale, and then multiplying by 100. An overall summary score was calculated as the average of the four domain scores. In this study, a summary score of <29 was defined as needing SC (26).

### Patient Data Management and Statistical Analysis

Patient responses from the KCCQ-12 were collected and managed using REDCap (Research Electronic Data Capture) electronic data capture tools hosted and supported by the Women and Children's Health Research Institute at the University of Alberta. REDCap is a secure, web-based application designed to support data capture for research studies, providing (1) an intuitive interface for validated data entry; (2) audit trails for tracking data manipulation and export procedures; (3) automated export procedures for seamless data downloads to common statistical packages; and (4) procedures for importing data from external sources (30). Continuous variables were expressed as medians with their respective interquartile ranges (IQRs). Categorical variables were expressed as the total number in each category, and the corresponding percentage of the study population they represented was calculated. All statistical analyses were performed using SPSS Statistics version 25 (IBM, NY, USA). Two-sided Fisher's exact test and two-tailed Mann Whitney test were used for categorical and continuous data, respectively.

## Results

### Patient-Reported Outcome Measures at Baseline

**Figure 1:**
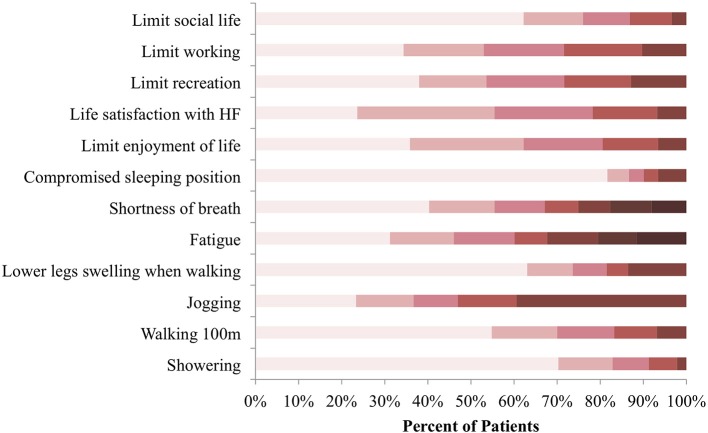
KCCQ-12 response distribution at baseline. Symptom burden, physical and social limitations, as well as quality of life impairment were reported in reference to the preceding 2 weeks before consultation. Colors categorize responses in terms of how limited patients were in the listed activities, with darker shades representing extreme limitation and lighter shades representing minimal to no limitation *N* = 456.

A total of 456 patients consented to take part in the study between February 6, 2018 and October 2, 2018 and fully completed the KCCQ-12. Symptom frequency, physical and social limitations, and quality of life impairment as a result of HF were assessed (Figure 1). Jogging or hurrying was affected the most by HF, with approximately 40% of patients indicating that they were “extremely limited” in the activity. Orthopnea was the least experienced by the majority of patients. More than 80% of participants indicated not having to sleep sitting up on a chair or with at least 3 pillows because of shortness of breath in the 2 weeks preceding consultation.

### Prevalence of SC Needs

**Figure 2:**
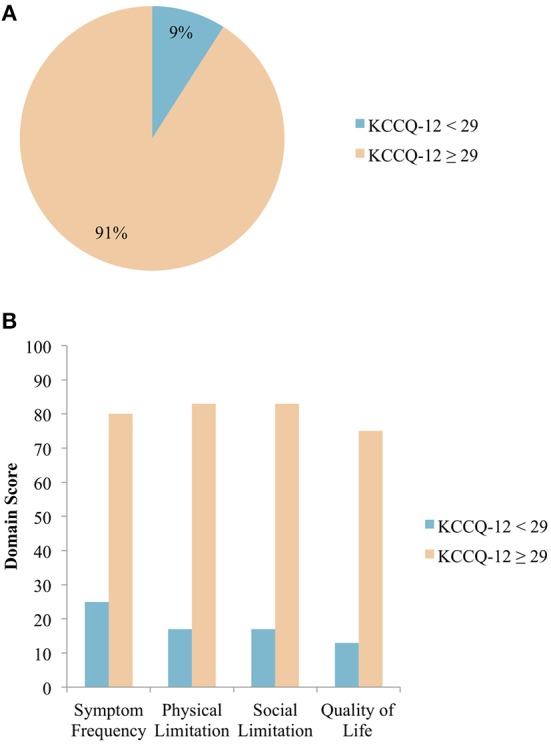
**(A)** Distribution of patients with HF requiring SC, grouped by KCCQ-12 summary score. Using a KCCQ-12 summary score cut-off of <29, 9% of patients with HF require SC *N* = 456. **(B)** Median domain scores in patients with and without SC needs. Median scores across the 4 domains as assessed by the KCCQ-12 are shown, with patients grouped according to the chosen KCCQ-12 cut-off score of <29 *N* = 456.

Of the 456 patients who completed all questions of the KCCQ-12, 41 (9%) had SC needs using the cut-off KCCQ-12 summary score of <29 (Figure 2A). The median scores for each of the 4 domains were calculated for patients with and without SC needs (Figure 2B). Patients with SC needs (KCCQ-12 score <29) reported higher symptom burden, as evidenced by these patients having lower scores for all 4 domains assessed by the KCCQ-12. The median KCCQ score for patients with KCCQ <29 and KCCC ≥ 29 was 21 and 76 (*p* < 0.0001), respectively.

### Patient Baseline Clinical Characteristics

The baseline demographic and clinical characteristics of patients were similar between the 2 groups (Table 1). However, NYHA classification distribution was significantly different between the 2 groups (*p* < 0.0001), with 76% of KCCQ-12 <29 patients belonging to NYHA class III and 55% of KCCQ-12 ≥ 29 patients belonging to NYHA class II. AHA staging of HF also showed significantly different distributions. KCCQ-12 <29 patients were either stage C (76%) or stage D (24%), while KCCQ-12 ≥ 29 patients were either stage B (8%) or stage C (92%). In terms of medical history, depression was significantly associated with KCCQ-12 <29 patients compared to KCCQ-12 ≥ 29 patients (*p* < 0.05). There were no significant differences observed for laboratory values or echocardiographic parameters. Both groups received similar medical therapies, except for mineralocorticoid receptor antagonists (MRAs), with 62% of KCCQ-12 ≥ 29 patients having received MRAs while only 44% of KCCQ-12 <29 patients were on this medication.

**Table 1:** Clinical characteristics of patient cohorts in this study.

**Criteria**	**KCCQ <29(*n* = 41)**	**KCCQ ≥ 29(*n* = 415)**	***P-*value**
**Age, years**	65 (59–78)	67 (58–77)	0.5890
**Female sex**	13 (32)	126 (30)	0.8600
**SBP, mmHg**	119 (103–131)	121 (110–135)	0.2843
**BMI, kg/m**^**2**^	32 (26–40)	30 (26–35)	0.1924
**NYHA class**			**<0.0001**
Class I	0 (0)	100 (24)	
Class II	6 (15)	230 (55)	
Class III	31 (76)	84 (20)	
Class IV	4 (10)	1 (0)	
AHA stage			**<0.0001**
Stage B	0 (0)	35 (8)	
Stage C	31 (76)	380 (92)	
Stage D	10 (24)	0 (0)	
**Medical history**			
Previous HF diagnosis	41 (100)	415 (100)	>0.9999
HF diagnosis >2 years	26 (63)	314 (76)	0.0927
HF hospitalization in preceding 6 months	11 (27)	104 (25)	0.8507
Hypertension	34 (83)	297 (72)	0.1431
Myocardial infarction	23 (56)	174 (42)	0.0982
Atrial fibrillation	14 (34)	196 (47)	0.1390
TIA/stroke	6 (15)	70 (17)	0.8289
Peripheral arterial disease	0 (0)	16 (4)	0.3817
Diabetes	19 (46)	167 (40)	0.5062
COPD	15 (37)	121 (29)	0.3709
Depression	17 (41)	102 (25)	**0.0248**
Cancer	10 (24)	63 (15)	0.1768
ICD/CRT-D	19 (46)	188 (45)	>0.9999
**Discharge medication**			
ACEi/ARB/sacubitril/valsartan	35 (85)	388 (93)	0.1033
Beta-blocker	37 (90)	385 (93)	0.5318
MRA	18 (44)	256 (62)	**0.0302**
Digoxin	2 (5)	42 (10)	0.4073
**Laboratory**			
BNP, pg/mL	374 (197–966)	304 (127–732)	0.4309
Na^+^, mmol/L	138 (136–141)	139 (137–141)	0.1189
eGFR, mL/m in/1.73 m^2^	51 (38–79)	63 (45–80)	0.1324
Hb, g/L	134 (110–149)	136 (125–149)	0.2166
**Echocardiography**			
EF ≤ 50%	18 (100)	166 (85)	0.1417
EF, %	32 (27–42)	34 (28–43)	0.5513
LVIDD/BSA, mm/m^2^	27 (24–30)	28 (25–31)	0.5614

*SBP, systolic blood pressure; BMI, body mass index; NYHA, New York Heart Association; AHA, American Heart Association; HF, heart failure; TIA, transient ischemic attack; COPD, chronic obstructive pulmonary disease; ICD, implantable cardioverter defibrillator; CRT-D, cardiac resynchronization therapy defibrillator; ACEi, angiotensin-converting enzyme inhibitor; ARB, angiotensin receptor blocker; MRA, mineralocorticoid receptor antagonist; BNP, B-type natriuretic peptide; Na^+^, sodium; eGFR, estimated glomerular filtration rate; Hb, hemoglobin; EF, ejection fraction; LVIDD, left ventricular internal diameter in diastole; BSA, body surface area. Bold values indicate significance*.

### Current Use of SC Services

**Figure 3:**
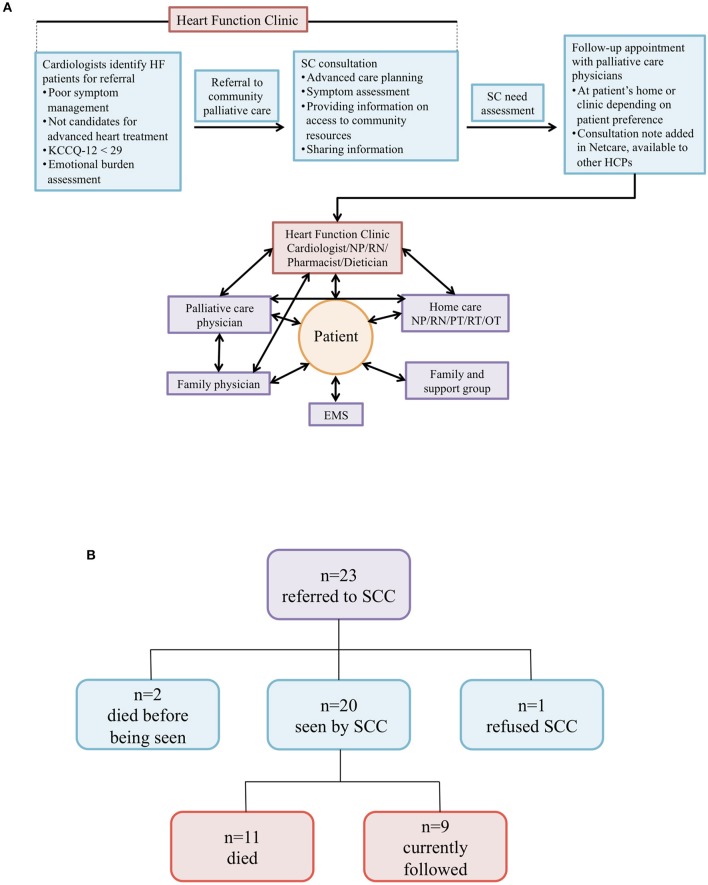
**(A)** Supportive care model for patients with HF. Schematic model of SC integrated into routine care for patients with HF at the Heart Function Clinic. HF, heart failure; SC, supportive care; HCPs, health care providers; NP, nurse practitioner; RN, registered nurse; PT, physical therapist; RT, respiratory therapist; OT, occupational therapist; EMS, emergency medical services. **(B)** Overview of heart failure patients referred to SCC. Twenty-three patients were initially referred to our SC clinic (SCC). Shown are the various outcomes we observed for these patients.

Twenty-three patients were referred to our newly established SC clinic (SCC) (Figure 3A). The median age of patients with HF referred to SC was 84 years with 83% of patients being male (Table 2). The majority of patients had emergency department visit(s) or hospitalization(s) in the preceding 6 months prior to SC referrals due to the worsening of HF. Furthermore, 83% of patients were on angiotensin-converting enzyme inhibitors (ACEis) or angiotensin receptor blockers (ARBs), and 91% were on beta-blockers. Only 52% were on MRAs due to poor renal function. The median B-type natriuretic peptide (BNP) value was 664 (IQR: 351-1132) pg/mL. All 23 patients displayed various co-morbidities such as coronary artery disease, renal failure and musculoskeletal abnormalities, among others (Table S1). Of those referred, 2 died before being seen, 1 refused SC due to accessibility concerns, and 20 were seen by the SCC team (Figure 3B). Of these 20 patients, 11 died and 9 patients are currently being followed as of June 7, 2019. Of the 11 patients that died, 2 died at the emergency department, 1 at the internal medicine unit, 5 at home, and 3 at hospital palliative care units.

**Table 2:** Clinical characteristics of patients referred to supportive care services.

**Demographics**		**Co-morbidities**		**Discharge medication**		**Laboratory**	
**Total number**	23									
**Age, years**	84 (77–88)	Hypertension	18 (78)	ACEi/ARB	19 (83)	BNP, pg/mL	664 (351–1132)
**Sex**		Myocardial infarction	13 (57)	Beta-blocker	21 (91)	Na^+^, mmol/L	139 (137–142)
Male	19 (83)	Atrial fibrillation	13 (57)	MRA	12 (52)	eGFR, mL/m in/1.73 m^2^	39 (25–55)
Female	4 (17)	TIA/stroke	4 (17)	Digoxin	3 (13)	Hb, g/L	118 (90–125)
**SBP, mmHg**	115 (105–134)	Peripheral arterial disease	5 (22)				
**BMI, kg/m**^**2**^	27 (25–29)	Diabetes	13 (57)				
**NYHA class**		COPD	12 (52)				
Class II	6 (26)	Depression	8 (35)				
Class III	17 (74)	Cancer	9 (39)				
**AHA stage**		ICD/CRT-D	12 (52)				
**Stage D**	23 (100)						
**Years of HF Dx**	4 (2–6)						
**ED/Hosp**	19 (83)						

*SBP, systolic blood pressure; BMI, body mass index; NYHA, New York Heart Association; AHA, American Heart Association; HF, heart failure; Dx, diagnosis; ER/Hosp, emergency department visits or hospitalization in the preceding 6 months; TIA, transient ischemic attack; COPD, chronic obstructive pulmonary disease; ICD, implantable cardioverter defibrillator; CRT-D, cardiac resynchronisation therapy defibrillator; ACEi, angiotensin-converting enzyme inhibitor; ARB, angiotensin receptor blocker; MRA, mineralocorticoid receptor antagonist; BNP, B-type natriuretic peptide; Na^+^, sodium; eGFR, estimated glomerular filtration rate; Hb, hemoglobin*.

Of the 23 patients referred to SC, KCCQ-12 summary scores were available for 17 patients (Table 3). Of those 17 patients, 6 (35%) had KCCQ-12 summary scores <29. The median score for 17 patients referred to SCC was 30 compared to 73 for the entire cohort (*p* < 0.0001). In terms of outcomes, 100% of patients referred to SC were referred to home care services. Of the 20 patients seen by SC physicians, 11 (55%) changed their goals of care (GOCs) from medical care and interventions focused on cure or control of illness to medical care and interventions focused on symptom control and comfort. Cardiac device deactivation was discussed with all patients and 1 had their device deactivated. Some patients expressed the wish to have their devices deactivated but passed away before the process was completed (data not shown). The median number of emergency department visit/hospitalization post-SC consultation was 1 (IQR: 0-2). The median survival time after SC initiation is 3 months, with patients spending anywhere from less than a month to 11 months before dying.

**Table 3:** KCCQ summary scores and clinical outcomes for patients referred to supportive care services.

**No**	**KCCQ SS**	**SC consultation**	**Death**	**ED/Hosp**	**Time to death (mo)**	**Location of death**	**Homecare referral**	**GOC change**	**ICD deactivation**
1	NA*	Feb, 18	Apr, 18	1	2	Hospital palliative care unit	Yes	M1 -> C1	No
2	NA*	Mar, 18	Jun, 18	0	3	Hospital palliative care unit	Yes	M1 -> C1	NA
3	NA*	Mar, 18	Aug, 18	2	5	Emergency department	Yes	M1 -> C1	NA
4	NA*	Apr, 18	Mar, 19	3	11	Home	Yes	M1 -> C1	NA
5	NA*	Apr, 18	NA	7	NA	NA	Yes	M1	NA
6	NA*	May, 18	Sep, 18	4	4	Home	Yes	C1	NA
7	62	Jun, 18	Jul, 18	1	1	Emergency department	Yes	R1	No
8	29	Jul, 18	Dec, 18	1	5	Hospital palliative care unit	Yes	M1 -> C1	NA
9	44	Sep, 18	NA	1	NA	NA	Yes	M1	No
10	18	Passed away before being seen	Sep, 18	NA	NA	NA	NA	NA	No
11	84	Nov, 18	NA	2	NA	NA	Yes	M1	No
12	14	Oct, 18	NA	1	NA	NA	Yes	R3 -> M1	No
13	30	Passed away before being seen	Nov, 18	NA	NA	NA	NA	NA	NA
14	6	Refused due to accessibility concerns	NA	NA	NA	NA	NA	NA	No
15	57	Oct, 18	Jan, 19	1	3	Home	Yes	M1 -> C1	No
16	58	Dec, 18	Jun, 19	0	6	Home	Yes	M1	NA
17	30	Jan, 19	NA	2	NA	NA	Yes	R3	NA
18	33	Oct, 18	NA	0	NA	NA	Yes	M1	No
19	69	Feb, 19	NA	0	NA	NA	Yes	R2	No
20	22	Mar, 19	Apr, 19	2	1	Hospital internal medicine unit	Yes	R1 -> M1	NA
21	23	Apr, 19	Apr, 19	0	0	Home	Yes	M1 -> C1	NA
22	75	Apr, 19	NA	0	NA	NA	Yes	M1 -> C1	Yes
23	10	Jun, 19	NA	0	NA	NA	Yes	R1 -> M1	No

SS, summary score; SC, supportive care; ED/Hosp, emergency department or hospitalization since SC initiation; GOC, goals of care; ICD, implantable cardioverter defibrillator; NA, not applicable. NA^*^, referred to SC before KCCQ.

## Discussion

While the symptom burden experienced by patients with HF significantly impairs their quality and enjoyment of life (9, 12, 31), it is challenging to identify which patients should be referred to SC (32, 33). The KCCQ-12 can bridge this gap to ensure that more patients with HF will benefit from SC (16, 26, 34). From patient baseline clinical data, traditional markers for HF such as estimated GFR, ejection fraction or BNP do not clearly identify which patients will subsequently need SC. Patients with SC needs were, however, more symptomatic. These results illustrate the concept of disease vs. illness. While HF can be objectively evaluated by certain physical and biochemical parameters, the responses of patients to disease and how it affects their lives are often individual and subjective experiences. For instance, we found that depression was associated with patients with SC needs. As such, incorporating both disease and illness perspectives into routine patient care facilitates the model of patient-centered care and will help improve individual patient health outcomes (35). To be representative and prevent bias we included all patients including those at AHA stage B (8%) in our study cohort (Table 1). Importantly, we wanted to assess our patients' quality of life and not rely solely on the absence or presence of HF symptoms since quality of life can be impaired by other co-morbidities. Using the cut-off KCCQ-12 of <29, Campbell et al. (26) identified 27% of patients with SC needs, while we identified 9%. Compared to our HF patient population, the patient cohort in the Campbell et al. study was older and had more co-morbidities, and generally involved a sicker in-patient population (26, 36, 37).

Our HFC provides comprehensive multidisciplinary care for patients with HF. Our team consists of cardiologists, nurse practitioners, registered nurses, dieticians and pharmacists who specialize in HF. When patients are referred to the HFC, a complete clinical assessment is performed. Patients with HF with poor symptom management and who are not candidates for advanced heart treatments such as ventricular assist device implantation or cardiac transplantation are referred to community palliative care physicians for SC consultation. At the consultation, palliative care physicians assess SC need and schedule a follow-up appointment if required. Our HFC then coordinates the care for the patient with other health care providers, e.g., palliative care physicians, family physicians and Edmonton home care service providers, to help deliver a comprehensive care plan. The main source of support for patients with HF in the community would be their family physicians or nurse practitioners along with home care service providers. Despite its great potential to improve patient quality of life, SC remains an under-utilized resource for patients with HF. Integration of additional screening tools such as the KCCQ-12 could improve our current model of care. Some patients with SC referrals had KCCQ summary scores ≥29 suggesting that although the KCCQ-12 is a useful tool, it may not completely capture the full spectrum of HF. Indeed, in this cohort, only 6 out of 17 patients (35%) had KCCQ-12 scores <29 clearly showing that additional assessment tools are needed in this patient population. Results from our study support the use of KCCQ-12 as an additional tool to assess SC needs in ambulatory HF patients, but it should always be used in conjunction with clinical judgment by physicians. Given the challenges in identifying patients with HF who are eligible for SC, additional SC questionnaires may be of use in clinical practice. Indeed, several standard palliative questionnaires including the Edmonton Symptom Assessment Scale and the Palliative Performance Scale demonstrate a modest correlation with the KCCQ (11).

Clinical outcomes for the 23 patients referred to SC services have shown positive results. Home care services provide patients with daily assistance and support, which eases the burden on them as well as their caregivers. Discussions surrounding GOCs provide patients the opportunities to align their care with what they feel is important. Also, these help alleviate a financial burden on the health care system by allowing for the redistribution of a limited set of resources to treat other patients who may instead need them. In terms of location of death, the majority of patients passed away at home or at the hospital palliative care unit. This is a favorable shift since patients are usually surrounded by family members and/or hospice care providers in these locations.

A large barrier that exists in supporting patients with HF is disease perception by patients and their families. Discussions about disease burden, prognosis, advance care planning, as well as patient GOCs are essential in the care of patients with HF. It is also intuitive that the management of these patients require a multidisciplinary team of physicians and allied health colleagues. We recognize that the small patient population at our SCC potentially reduces generalizability of the outcome data. However, this only highlights the imbalance between the demand and supply of SC for patients with HF, an issue that the field is currently experiencing. Another possible limitation of our study is that KCCQ-12 scores were not available for the 6 patients referred to our SCC, which makes evaluation of the KCCQ-12's usefulness for identifying SC need more challenging. Despite these barriers, the incorporation of SC into routine care has the potential to improve the quality of life for patients with HF. Initiatives to facilitate this effort should therefore be supported and promoted.

## Data Availability Statement

The raw data supporting the conclusions of this manuscript will be made available by the authors, without reservation, to any qualified researcher.

## Ethics Statement

The studies involving human participants were reviewed and approved by Health Research Ethics Board at the University of Alberta. The patients/participants provided their written informed consent to participate in this study.

## Author Contributions

GO, MM, and BC conceived and designed the study. GO, BC, JE, and ID supervised the study. QN, KW, AN, and DC-S performed data collection. QN performed data and statistical analysis. QN and GO wrote the manuscript. All authors contributed to manuscript revision, read, and approved the submitted version.

### Conflict of Interest

The authors declare that the research was conducted in the absence of any commercial or financial relationships that could be construed as a potential conflict of interest.
