# Supplementary material for: Screening and Initiating Supportive Care in Patients With Heart Failure
Source: Front Cardiovasc Med. 2019 Oct 22;6:151. doi: 10.3389/fcvm.2019.00151 (PMC6817607; doi:10.3389/fcvm.2019.00151)
Supplement: Supplementary file 1 [file Data_Sheet_1.PDF]

Supplementary Material

Table S1. Clinical characteristics of 23 patients referred to supportive care clinic (SCC)

#	Age	Sex	SBP	BMI	NYHA	HF Dx Year	ED/ Hosp	HTN	MI	AF	STR	PAD	DM	COPD	Dep	Cancer	ICD/ CRT-D	ACEi /ARB	βB	MRA	Dig	BNP pg/mL	Na+ mmol/L	eGFR mL/min/1.73m^2	Hb g/L	Other Co-mobidities
1	84	M	158	24	3	2016	3	Y	Y	Y	N	Y	Y	N	Y	N	Y	N	Y	N	N	419	141	52	125	CAD, renal failure, dyslipidemia, anemia, severe gastritis and Candida sophagitic, bundle branch block
2	98	F	108	31	3	2014	1	Y	N	N	N	N	Y	N	Y	N	N	Y	N	N	N	212	126	9	118	Aortic stenosis, dyslipidemia, right thoracentesis, CKD
3	93	M	114	22	3	2010	2	N	Y	Y	N	N	Y	Y	Y	Y	N	Y	Y	N	N	1148	141	21	90	Ischemic cardiomyopathy, GERD, diverticulosis
4	91	F	140	31	3	2017	2	Y	Y	N	N	N	Y	Y	Y	N	N	Y	Y	Y	N	1023	143	63	81	GERD, osteoporosis, scoliosis, cognitive impairment, anemia, pneumonia
5	87	M	115	25	2	2017	0	Y	N	Y	N	N	Y	N	Y	N	N	N	Y	N	N	NA	137	55	117	AVM of colon, varicose veins, CAD, anemia, GI bleed, hypothyroidism, dyslipidemia
6	93	M	102	26	3	2016	1	N	N	Y	N	N	N	Y	N	Y	N	N	N	N	Y	253	142	55	79	Pneumonia, lung nodules, bundle branch block, prostate hyperplasia, left lip squamous cell carcinoma, macular degeneration
7	59	M	114	21	3	2017	4	N	Y	N	N	N	Y	Y	N	Y	Y	Y	Y	Y	Y	3100	137	97	124	Dilated ischemic cardiomyopathy, renal cell carcinoma
8	86	M	116	27	3	2015	2	N	N	Y	N	N	N	N	N	N	N	Y	Y	N	N	1337	137	25	123	Chronic renal impairment, albuminuria, peripheral sensosy neuropathy, OSA
9	34	F	90	22	2	2012	1	N	N	N	N	N	Y	N	N	N	Y	Y	Y	Y	Y	NA	NA	NA	NA	Cardiomyopathy, osteoporosis, dyslipidemia, seizure, myopathy, proteinuria, deafness, optic atrophy, microalbuminuria, corpus callosum agenesis, pneumonia, anemia, gout, holoprosencephaly
10	70	M	90	32	3	2012	6	Y	Y	N	N	Y	N	Y	Y	Y	Y	Y	Y	Y	N	3840	138	61	78	CAD, DVT, ischemic cardiomyopathy, AML, dyspilidemia, anxiety, PAD
11	78	M	135	44	3	2015	0	Y	N	N	N	N	Y	Y	N	N	Y	Y	Y	N	N	78	143	24	118	CAD, AV block, dyslipidemia, gout, insomnia, obesity hypoventilation syndrome, obesity, cognitive impairment
12	81	M	96	28	2	2010	1	Y	Y	N	N	N	N	Y	N	N	Y	Y	Y	Y	N	558	142	51	130	Lumbar spondylotic stenosis, VT, dilated cardiomyopathy, bundle branch block, hypothyroidism, GERD, osteoarthritis, dyslipidemia, OSA, renal failure
13	83	M	155	29	3	2017	2	Y	Y	Y	Y	Y	N	Y	N	N	N	Y	Y	N	N	1115	141	18	87	Aortic stenosis, OSA, hypothyroidism, gout, anemia
14	89	M	122	25	3	2014	0	Y	N	Y	N	N	Y	N	N	N	Y	Y	Y	N	N	NA	NA	NA	NA	Dyslipidemia, CAD, renal impairment, tachycardia induced cardiomyopathy, aortic root dilation
15	73	M	93	23	3	2016	1	Y	N	Y	N	Y	Y	N	Y	Y	Y	N	Y	Y	N	664	128	25	98	Prostate cancer, hypothyroidism, tubular adenoma of colon, gout, inguinal hernia, hematuria, myositis, sarcoidosis, CAD, prostatitis, CKD, hyponatremia, iliac artery dissection
16	90	M	143	26	3	2018	2	Y	N	N	N	N	N	N	N	Y	N	Y	Y	Y	N	1543	144	48	134	Prostate cancer, squamous cell carcinoma, basal cell carcinoma, aortic stenosis, dementia, GERD, diverticulosis
17	76	F	133	28	2	2018	0	Y	N	N	N	N	N	N	N	N	N	Y	Y	N	N	NA	138	37	126	Hashimoto thyroiditis, CKD, gout, DVT, fatty liver
18	79	M	109	29	3	2013	1	Y	Y	Y	N	Y	N	N	N	N	Y	Y	Y	Y	N	972	134	44	107	CAD, dyslipidemia, DVT, CKD, hypothyroidism, PAD, hemochromatosis
19	81	M	147	27	2	2010	4	Y	Y	Y	Y	N	Y	Y	N	N	Y	Y	Y	Y	N	482	136	67	133	VT, ischemic cardiomyopathy, AIVR, complete AV block, CAD, dyslipidemia, DVT, COPD, Barret esophagus gastritis, gout, osteroarthritis, left inguinal hernia
20	70	M	118	29	3	2015	2	Y	Y	Y	Y	N	N	Y	Y	Y	N	Y	Y	Y	N	480	144	34	83	CAD, AF, HTN, dyslipidemia, stroke, gastric angiodysplasia, pulmonary nodules, squamous cell carcinoma of the anal canal
21	87	M	113	29	3	2018	1	Y	Y	Y	Y	N	N	N	N	Y	N	Y	Y	Y	N	907	139	8	121	CAD, gout, thrombosis, stroke, prostate cancer, CKD, GERD, atrial flutter, AF, anemia, pulmonary hypertension, valvular disease
22	84	M	124	25	2	2014	3	Y	Y	N	N	N	Y	Y	N	N	Y	Y	Y	N	N	282	138	37	156	CAD, ventricular tachycardia, hypothyroidism, GERD, gout, hyperlipidemia, diverticular disease, vertigo
23	84	M	99	28	3	2017	3	Y	Y	Y	N	N	Y	Y	N	Y	Y	Y	Y	Y	N	907	139	8	121	CAD, dyslipidemia, osteoarthritis, A fib, CKD, mitral regurgitation, tricuspid regurgitation, complete heart block

SBP, systolic blood pressure; BMI, body mass index; NYHA, New York Heart Association; HF, heart failure; Dx, diagnosis; ED/Hosp, emergency department or hospitalization in the preceeding 6 months; HTN, hypertension; MI, myocardial infarction; AF, atrial fibrillation; STR, stroke; PAD, peripheral arterial disease; DM, diabetes mellitus; COPD, chronic obstructive pulmonary disease; Dep, depression; ICD, implantable cardioverter defibrillator; CRT-D, cardiac resynchronisation therapy defibrillator; ACEi, angiotensin-converting enzyme inhibitor; ARB, angiotensin receptor blocker; βB, β blocker; MRA, mineralocorticoid receptor antagonist; Dig, digoxin; BNP, B-type natriuretic peptide; Na+, sodium; eGFR, estimated glomerular filtration rate; Hb, haemoglobin; CAD, coronary artery disease; CKD, chronic kidney disease; GERD, gastroesophageal reflux disease; AVM, arteriovenous malformation; GI, gastrointestinal; OSA, osteoarthritis; DVT, deep vein thrombosis; AV, atrioventricular; VT, ventricular tachycardia; AIVR, accelerated idioventricular rhythm.
